# Reports on the Hospitals of the United Kingdom

**Published:** 1909-12-04

**Authors:** Henry Burdett


### REPORTS
ON THE
### Hospitals of the United Kingdom.

By SIR HENRY BURDETT, K.C.B., K.C.V.O.

### POOR LAW INFIRMARIES.

### SOME GENERAL AND SPECIAL CONSIDERATIONS.

Poor Law infirmaries have quietly, but on the whole progressively, developed their methods of treatment and administration until the best of them are now entitled to rank as hospitals. It is of the first importance that everything which can be done should be done to make these Poor Law infirmaries efficient and up-to-date in all respects. They, of course, differ materially in relative efficiency. Some of them are of the Charles Dickens type, like the Salford Union Workhouse Infirmary, at Hope, which has far too long been allowed to shock the public conscience by the inefficiency and incapacity of the Board of Guardians, who have had it at their mercy. Some of them are highly efficient, like the Metropolitan Poor Law Asylums, that at Birmingham, and those of the Chorlton Union, Manchester, and Crumpsall Workhouse, Manchester. All these and several other institutions bear remarkable evidence to the enormous value of zealous personal service on the part of the educated gentlewomen who have had the energy and self-denial to accept the office of lady superintendent, and to devote their lives in making the Poor Law infirmary as efficient as possible. The conditions which some of these ladies have had to face have been lamentable, and Miss Bradley, who has, single-handed, held the flag of efficiency at the Hope Hospital with purposeful rectitude and has overcome all the machinations of a particularly offensive and cowardly opposition, should win the gratitude of everyone working in a reputable hospital and of those who have been pioneers and leaders in the reform movement in support of an adequate Poor Law provision for the sick. When all has been done—and it is much—that these ladies, backed up with zeal and energy, and assisted by medical superintendents of the higher type, can do, there remain a number of points to be settled which affect the public, and these the public ought to deal with effectually.

Anyone who inspects a Poor Law infirmary must be struck with the attitude and appearance of many of those to be met with in the day rooms. They have nothing to do apparently, and the hours must hang heavily upon their hands. The lady superintendents employ the women as much as possible in sewing, and much good work is accomplished. The men are engaged in assisting in the ward work, but here we hold that pauper labour should be dispensed with in Poor Law infirmaries so far as the wards and attendance upon the sick are concerned.

When everything that is being attempted is taken fully into account there remains a large number of convalescents in Poor Law infirmaries who would welcome occupation and relaxation. Useful work in abundance might be found for them, as it might be found for a great number of equally unfortunate persons who habitually reside in the workhouses, were it not for the action of the Labour Party in general and Trade Unions in particular. Why these bodies should make a set against the useful employment of the poor unfortunates in the care of the Poor Law, we know not. All we can say is that it cannot rest upon any solid ground, nor does it reflect honour either upon the intelligence or the humanity of those who are responsible for this policy. The actual numbers of unemployables under the Poor Law must constitute an unimportant total so far as employment is concerned, whilst acute misery and even suffering is inflicted upon a large number of the least fortunate members of the race by the enforcement of the existing policy of the Labour Party and Trades Unions. We hope that one result of the work done by the recent Poor Law Commission will be to adjust these matters and so secure justice for those who are least able to take care of themselves.

Another very important matter is the absence of any real vitalising interest in the sick in Poor Law infirmaries on the part of the residents in the districts where these institutions are situated. It would be of immense value if the people in the neighbourhood who have a little leisure, or have gardens, or are able to make themselves useful by taking part in providing concerts and other amusements for the benefit of the inmates, would throw themselves into the work of making the lot of the patients in Poor Law infirmaries happier, brighter, and more endurable, than it is at present. The very fact that the Poor Law infirmary is so neglected by the outside world should cause the silent and retiring and solitary of the better class to arouse themselves and make these institutions *their* care, so that the hearts of the lady superintendents, medical officers, and their staff, may be cheered, and the lot of the patients may be brightened, by a stream of outside sympathy, and the introduction of new and vigorous life, under proper regulations, into these hospitals. What is wanted is that the better class residents, or some of them, should decide to render in their days of health a measure of personal service in the cause of the sick by devoting some time each day to the patients in the neighbouring Poor Law infirmary. If this were once done, even in connection with one of the workhouse infirmaries—done, that is, not merely by lady visitors bringing a few books, getting up entertainments at Christmas, valuable as that work is, but by steady, purposeful organisation—the workhouse infirmary would be brightened and its influence for good increased to an extent at present not understood by the people at large. We can only suggest these points for the earnest consideration of all those whom they may concern.

There is one matter which should especially appeal to women and mothers—that is, the children's wards. There are a great number of children in each Poor Law infirmary, and at present their life is not made as bright and happy as it might be, for the lack of a few generous people to visit these wards occasionally and take care to organise an adequate supply of toys and other amusements so that the children may have a chance of being helped towards recovery in the pleasantest and best way. Heaps of people, we are confident, have sympathy with all children so circumstanced, but what we want nowadays is the exhibition of the spirit which impels residents in a district where a Poor Law infirmary is situated to write to the lady superintendent and ask her whether presents of toys and other things would be acceptable, and to offer their co-operation to organise means whereby an adequate supply of these accessories can be always available.

### XII.—CRUMPSALL WORKHOUSE INFIRMARY, MANCHESTER.

This infirmary was established in 1877, and for many years the nursing staff has been under the control of Miss Mary Girdlestone, the lady superintendent, one of the ablest of the trained nurses engaged in this work. She has effected wonders at Crumpsall, and the atmosphere of the whole institution is remarkable for its efficiency, as well as for the excellent spirit which prevails in every department under her charge. The walls of the wards are not plastered, but Miss Girdlestone has managed to provide for the upper parts of them to be colour-washed once every year, for the improvement of the quality of the beds and bedding, and the addition of such ward furniture as is absolutely essential. By these means and by the perfect cleanliness and order which is maintained throughout this immense hospital with its 1,400 beds and an average occupancy of 1,200, the general condition of the hospital is as inspiriting as it is remarkable.

### A Great Reform.

The former medical officer, Dr. Reynolds, did an immense service by organising a system of classification for the inmates of the Crumpsall Workhouse Infirmary which has had great influence for good, has increased the comfort of the patients, and improved the moral tone of the whole establishment. Classification, as we have contended for quite 30 years, has made for efficiency, discipline, comfort, and the moral improvement of each individual and of every ward. It has removed a multitude of evils which were at one time rampant in workhouse hospitals. All honour to Dr. Reynolds, whose system should everywhere be in force in establishments of this kind. He has left the service now, but his good works do follow him.

There is one matter which has been the subject of discussion in the columns of The Hospital recently which is of great importance to the efficiency of every workhouse infirmary and the proper treatment of its patients. That is, the question as to how far it is desirable to have a visiting medical staff attached to these institutions. In our view, after going very closely into the matter, the best system is to have a medical superintendent in charge of every workhouse infirmary, and in addition, to have attached to each one of these hospitals a visiting surgeon of repute. This latter officer should have charge of the surgical wards, and he should devote himself actively to the surgical cases so that there may be no patient who would be benefited by an operation who has not the advantage of such treatment. Every operation should thus be performed under the best conditions and afford each patient the best prospect of a successful issue. We are quite certain that it is undesirable that any portion of the staff, or indeed any portion of the Poor Law Infirmary, should be under the direction of the Master of the workhouse. The appointment of a medical superintendent in charge of the whole administration of a Poor Law Infirmary obviates the necessity to have a Master, and if it is thought advisable by the Guardians to have a responsible layman in charge of the stores and the male servants, such an officer should be under the direct control of the medical superintendent as head of the establishment.

### Minor, but Important, Matters.

Miss Girdlestone is to be congratulated in having secured a separate kitchen in connection with the food supply of the nurses and staff. This is a very important reform because it adds greatly to the comfort of those immediately concerned, and also secures economical working. We should like to know whether separate accounts of the actual cost of working the kitchen for the nurses and staff, and also for the general kitchen for the rest of the establishment, are kept, and to see a copy of them. Though parts of the buildings are old, many of them have been made exceedingly attractive, comfortable, and pretty in appearance. It may seem at the first glance well nigh impossible to secure such a result when the ward walls consist of rough bricks indifferently set and unplastered. Miss Girdlestone has overcome the difficulties presented by this state of affairs, and has shown that good paint put upon bricks of this class can be made to look presentable and even attractive. The fittings are old and the wash-hand basins, or most of them, require renewal. We are of opinion that white basins with slate tops and the best taps are on the whole the most suitable appliances for the patients' lavatories in a workhouse infirmary. In the ward kitchens, instead of wood, or porcelain, or slate, we think experience has proved that block tin is the best material to use, for it can be kept bright and attractive under all conditions. Block tin obviates the dirty, repellent appearance of the scullery incline boards when they consist of wood only. At Crumpsall, as at many other places, the Rufford baths have been in use for very many years, and have proved an excellent investment by wearing so well. They still present an appearance after 20 years' wear at least which, on the whole, is fairly good. A great feature of this Poor Law Infirmary, and of others we have visited, is the perfect cleanliness which has been attained, and the wonderful brightness of the china, plate, and everything in which food is served. So exceptionally high is the standard of efficiency in this respect that it is remarkable, for it seems to us even to exceed that attained by some of the voluntary hospitals. In the operation theatre we found linoleum in use on the floor. Whether this is a good or bad thing remains to be proved, but, seeing that the linoleum in question is cracked, it has proved already to be not aseptic, and is so condemned for such a use. The Nurses' Home is good, but, in our opinion, a few more chairs of a very comfortable kind should be supplied to the relaxation-rooms.

### A Great Superintendent.

We cannot conclude this report without expressing a sense of the credit which is due to Miss Girdlestone and her staff for the high quality of the work they are doing without public recognition or reward. It is remarkable in these days that it should be done without practically any knowledge in the outside world of the work or of its excellence. The attitude of indifference which the public exhibit towards Poor Law Infirmaries is not only regrettable but reprehensible. These great institutions are not dust-heaps but hospitals, and hospitals of a type which deserve public recognition. They are well worthy the personal attention and sympathy of the residents in the districts where they are situated, and we hope that many such residents will bestir themselves, place themselves in communication with the Lady Superintendent, and decide to devote a measure of their time to the work of making the lot of the inmates less monotonous and irksome than it is at present.

**The Leicester Infirmary** will be reported on next week.

